# Comprehensive Pan-Cancer Analysis of KIF18A as a Marker for Prognosis and Immunity

**DOI:** 10.3390/biom13020326

**Published:** 2023-02-08

**Authors:** Ting Liu, Kun Yang, Jiamin Chen, Liming Qi, Xingang Zhou, Peng Wang

**Affiliations:** Department of Pathology, Beijing Ditan Hospital, Capital Medical University, No. 8 Jing Shun East Street, Chaoyang District, Beijing 100015, China

**Keywords:** KIF18A, prognosis, immune cell infiltration, pan-cancer

## Abstract

KIF18A belongs to the Kinesin family, which participates in the occurrence and progression of tumors. However, few pan-cancer analyses have been performed on KIF18A to date. We used multiple public databases such as TIMER, The Cancer Genome Atlas (TCGA), Genotype-Tissue Expression (GTEx), and Human Protein Atlas (HPA) to explore KIF18A mRNA expression in 33 tumors. We performed immunohistochemistry on liver cancer and pancreatic cancer tissues and corresponding normal tissues to examine the expression of KIF18A protein. Univariate Cox regression and Kaplan–Meier survival analysis were applied to detect the effect of KIF18A on overall survival (OS), disease-specific survival (DSS), and progression-free interval (PFI) of patients with these tumors. Subsequently, we explored KIF18A gene alterations in different tumor tissues using cBioPortal. The relationship between KIF18A and clinical characteristics, tumor microenvironment (TME), immune regulatory genes, immune checkpoints, tumor mutational burden (TMB), microsatellite instability (MSI), mismatch repairs (MMRs), DNA methylation, RNA methylation, and drug sensitivity was applied for further study using the R language. Gene Set Enrichment Analysis (GSEA) was utilized to explore the molecular mechanism of KIF18A. Bioinformatic analysis and immunohistochemical experiments confirmed that KIF18A was up-regulated in 27 tumors and was correlated with the T stage, N stage, pathological stage, histological grade, and Ki-67 index in many cancers. The overexpression of KIF18A had poor OS, DSS, and PFI in adrenocortical carcinoma (ACC), kidney renal clear cell carcinoma (KIRC), kidney renal papillary cell carcinoma (KIRP), brain lower-grade glioma (LGG), liver cancer (LIHC), lung adenocarcinoma (LUAD), and pancreatic cancer (PAAD). Univariate and multivariate regression analysis confirmed KIF18A as an independent prognostic factor for LIHC and PAAD. The mutation frequency of KIF18A is the highest in endometrial cancer. KIF18A expression levels were positively associated with immunocyte infiltration, immune regulatory genes, immune checkpoints, TMB, MSI, MMRs, DNA methylation, RNA methylation, and drug sensitivity in certain cancers. In addition, we discovered that KIF18A participated in the cell cycle at the single-cell level and GSEA analysis for most cancers. These findings suggested that KIF18A could be regarded as a latent prognostic marker and a new target for cancer immunological therapy.

## 1. Introduction

Cancer has become the main cause of death worldwide and is a public health issue. The occurrence of cancer is a complicated process that requires the involvement of many signaling pathways and genes. Recent research has revealed that tumor occurrence, progression, recurrence, and metastasis are closely related to the tumor microenvironment (TME). Tumor immunity has a major part to play in tumor recurrence and poor prognosis, so it is necessary to study its related mechanisms.

Kinesin is a microtubule-associated motor involved in intracellular material transport and cell division [1,2,3]. Kinesins are divided into three classes: N-type kinesins, M-type kinesins, and C-type kinesins. Human kinesins consist of 15 subfamilies, including 45 members [4]. KIF18A belongs to the Kinesin family; it binds to microtubules and moves along them using energy from ATP hydrolysis. It is related to the localization of membrane organelles and protein complexes, as well as the maintenance of microtubule dynamics and mitosis [5,6]. A dysfunctional expression of KIF18A results in abnormal sister chromatid segregation during mitosis, resulting in tumor development [7]. Some studies showed that RNA interference inhibited KIF18A expression in cells, and cells were blocked at G2/M, indicating that KIF18A blocks cells and regulates the cell cycle [8]. KIF18A expression is low in normal tissues and high in solid tumors, including breast cancer (BRCA) [9,10,11], hepatocellular carcinoma (HCC) [12,13] and lung adenocarcinoma (LUAD) [14,15], colorectal cancer(COAD) [16], renal cell carcinoma (RCC) [17], and head and neck squamous cell carcinoma (HNSCC) [18]. KIF18A accelerates the invasion and migration of HNSCC by activating the AKT signaling pathway [18]. Zhang et al. discovered that the up-regulation of KIF18A in human breast cancers causes high malignancy, high metastasis rates, and poor prognosis [9]. KIF18A is overexpressed in esophageal carcinoma, and knockdown of KIF18A can reduce cell proliferation and migration and enhance radiosensitivity [19]. However, the majority of previous research focused only on the function of KIF18A in a certain cancer; pan-cancer analyses on the prognostic significance and biological function of KIF18A are rarely carried out. Our study systematically discusses the association between KIF18A and transcription level, clinical prognosis, TME, tumor mutational load (TMB), microsatellite instability (MSI), mismatch repair (MMR), and DNA and RNA methylation in 33 cancers, providing strategies and directions for future research on KIF18A-based anti-tumor therapy.

## 2. Materials and Methods

### 2.1. Gene Expression Analysis of KIF18A in Pan-Cancer

The TIMER database (http://cistrome.dfci.harvard.edu/TIMER/, accessed on 6 April 2022) is an analysis network of tumor immune cell infiltration. It can not only analyze the infiltration level of tumor immune cells but also the differential gene expression between tumor tissue and normal tissues [20]. Additionally, because some tumors in TCGA lack normal tissues, we acquired RNA-seq data for 33 cancers from The Cancer Genome Atlas (TCGA) database and Genotype-Tissue Expression (GTEx) database. The abbreviations of 33 types of tumors are shown in Table 1. Wilcoxon tests were utilized to analyze significant differences. Moreover, we investigated the protein expression level of KIF18A in some tumors using the Human Protein Atlas (HPA) database and determined the subcellular localization of KIF18A via indirect immunofluorescence microscopy (https://www.proteinatlas.org/search/KIF18A, accessed on 6 April 2022) [21].

### 2.2. Prognostic Analysis of KIF18A

The clinical information of 33 tumors was downloaded from the TCGA database. We selected overall survival (OS), disease-specific survival (DSS), and progression-free interval (PFI) to analyze the correlation between KIF18A expression and the 33 cancers’ prognoses. The patients were separated into high and low KIF18A groups according to the median KIF18A expression value with 50% cut-off high and 50% cut-off low [22]. Kaplan–Meier survival analysis and Cox regression were conducted to explore the correlation between KIF18A and survival prognosis using “survminer” and “survival” packages. Cox regression was visualized by the “forest plot”. To further explore the predictive power of KIF18A, a time-dependent ROC curve was also conducted using timeROC of R language.

### 2.3. Relationship between KIF18A and Clinicopathologic Features

Wilcoxon test was applied to explore the correlation between KIF18A expression and clinicopathologic features, including T stage, N stage, clinicopathological stage, histologic grade, and ki67 index. The ROC curve was performed to investigate the KIF18A diagnostics values in 33 kinds of tumors using the pROC package in R language.

### 2.4. Gene Mutation Analysis of KIF18A

cBioPortal (https://www.cbioportal.org/, accessed on 7 April 2022) is a web platform for analyzing tumor genomic characteristics in the KIF18A gene. We performed the pan-cancer mutation frequency analysis of KIF18A using this database [23]. The mutation alterations included mutation, amplification, and deep deletion.

### 2.5. Relationship between KIF18A Expression and Immunity

The R “GSVA” package was utilized to explore the relationship between KIF18A and 24 kinds of immune infiltrating cells by single-sample Gene Set Enrichment (ssGSEA) [24]. ESTIMATE is an algorithm that utilized expression data to evaluate stromal cells and immune cells in 33 tumors, which predicts TME based on immune and stromal scores. “ESTIMATE”package in R was utilized to study the association between KIF18A expression and stromal cells and immune cells of 33 cancers [25]. We then acquired heat maps of Spearman’s correlations between KIF18A and different types of immune cells. Furthermore, we investigated the association between KIF18A and immune regulatory genes, including major histocompatibility complexes (MHC), immunostimulators, chemokines, and chemokine receptors based on the TCGA database. Correlative studies on KIF18A expression with immune checkpoints were also discussed by Spearman analysis.

### 2.6. Correlational Research on KIF18A and TMB, MSI, and MMR

TMB is defined as the total number of base mutations per million cells in the tumor. It is generally believed that TMB can stimulate the production of tumor-specific and high-immunogen antibodies and is a novel target for predicting the effect of tumor immunotherapy [26]. MSI is caused by abnormal DNA MMR and leads to gene duplication disorder and tumor development, affecting tumor prognosis [27,28,29]. Correlation between KIF18A and TMB and MSI was conducted using Spearman correlation analysis. MMR is a genetic monitoring mechanism that identifies and repairs mismatched nucleotides during DNA replication to maintain genetic stability [30]. In addition, Spearman’s correlation was applied to study the relationship between KIF18A and DNA MMR proteins, including MLH1, MSH2, MSH6, PMS2, and EPCAM.

### 2.7. DNA and RNA Methylation Analysis of KIF18A

DNA methylation is a common form of epigenetic modification. Aberrant DNA methylation may exist in tumor cells [31]. We searched the UALCAN database (http://ualcan.path.uab.edu/, accessed on 7 April 2022) to explore KIF18A promoter DNA methylation levels in certain cancers to determine the differences between tumors and normal tissues. Shiny Methylation Analysis Resource Tool (SMART, http://www.bioinfo-zs.com/smartapp/, accessed on 7 April 2022) was applied to discuss the distribution of methylation probes in chromosomes [32]. MethSurv (https://Biit.cs.ut.ee/MethSurv/, accessed on 7 April 2022) is an online server for analyzing DNA methylation mode survival [33]. RNA methylation is an important epigenetic regulatory pathway and takes part in tumor genesis, development, and prognosis [34]. RNA methylation includes 6-methyladenosine(m6A), 5-methylcytidine(m5C), and 1-methyladenosine(m1A). We explored the correlation between KIF18A and regulatory proteins of m6A, m5C, and m1A using Spearman correlation analysis.

### 2.8. Single-Cell Functional Analysis of KIF18A

The Cancer single-cell state Atlas (CancerSEA, http://biocc.hrbmu.edu.cn/CancerSEA/, accessed on 8 April 2022) is an analytic tool for studying cancer cell functions at the single-cell level, containing 14 tumor-related cellular functions of 900 cancer cells from 25 cancers [35].

### 2.9. Drug Sensitivity Analysis

GSCALite (http://bioinfo.life.hust.edu.cn/web/GSCALite/, accessed on 8 April 2022) is a comprehensive platform for analyzing gene expression and drug sensitivity analysis.

### 2.10. Co-Expressed Genes and Enrichment Analysis of KIF18A

GeneMANIA (http://genemania.org/, accessed on 8 April 2022) is an online tool for exploring gene interactions and functions and identifying co-expressed genes [36]. Forty genes co-expressed with KIF18A were obtained through GeneMANIA. Gene Ontology (GO) and Kyoto Encyclopedia of Genes and Genomes (KEGG) enrichment were conducted on them using the R package “Cluster Profile”. Finally, we utilized Gene Set Enrichment(GSEA) to research the biological functions between the low and high expressions of KIF18A for pan-cancer using the R package “Cluster Profile”.

### 2.11. Immunohistochemistry (IHC)

Ten cases of liver cancer and ten cases of pancreatic cancer and their corresponding normal tissues were collected from the Department of Pathology, Beijing Ditan Hospital, Capital Medical University, for immunohistochemical staining. The study was authorized by local institutional review boards (batch number: NO.DTEC-KT2022-003-01), and written informed consent was obtained from all patients before surgery. The slides were deparaffinized, antigen repair was performed with antigen retrieval solution, endogenous peroxidase activity was blocked with 3% hydrogen peroxide, followed by goat serum to reduce non-specific staining. Subsequently, KIF18A rabbit polyclonal antibody (19245-1-AP, 1:100, Proteintech Company, IL, USA) was added to the slide and left overnight at 4 °C. The next day, sheep anti-rabbit IgG polymer (PV-6000, Zhongshan Jinqiao Biotechnology Company, Beijing, China) was added for half an hour at room temperature, followed by DAB for 3 min. Then, they were counterstained with hematoxylin and dehydrated and sealed with neutral glue. KIF18A protein expression in different cancers and normal samples was detected using IHC. KIF18A staining was localized in the cytoplasm. KIF18A protein expression was scored semi-quantitatively, which was equal to expression intensity multiplied by expression area. Five different fields were randomly selected and observed under a microscope (Nikon Company, Tokyo, Japan) at 200 magnification. The expression intensity was scored from 0 to 3, indicating negative, weakly staining (light yellow), moderately staining (light brown), and strongly staining (dark brown), respectively. Expression area was scored from 0 to 4, representing <5, 6–25, 26–50, 51–75, >75%, respectively. The degree of positive staining was defined: 1~3 as weak positive (+); 4–6 as moderately positive (++); 7–12 as strong positive (+++) [37].

### 2.12. Statistics Analysis

Wilcoxon test was applied to investigate KIF18A expression and its correlation with clinical characteristics according to the TCGA and GTEx database. Survival curves and logistic regression analysis were drawn using the R packages “survival” and “survminer”. The R package “Cluster Profile” in the R software (Version4.2.1, R Foundation for Statistical Computing, Beijing, China) was used to analyze GO, KEGG pathway, and GSEA enrichment. Spearman correlation analysis estimated the correlation between KIF18A and TMB, MSI, MMR, and RNA methylation; *p* < 0.05 indicates statistical significance. 

## 3. Result

### 3.1. The Expression of KIF18A and Diagnosis Value in Pan-Cancer

**Figure 1:**
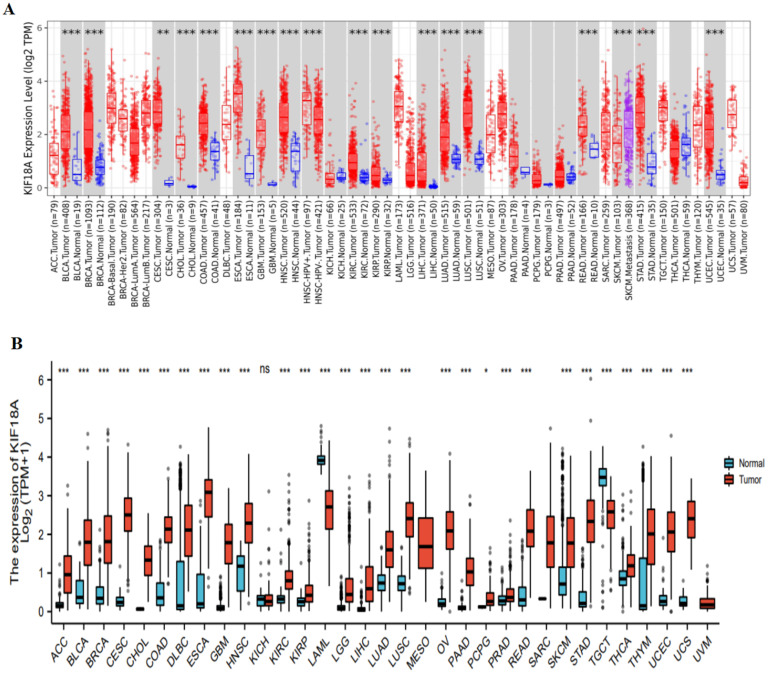

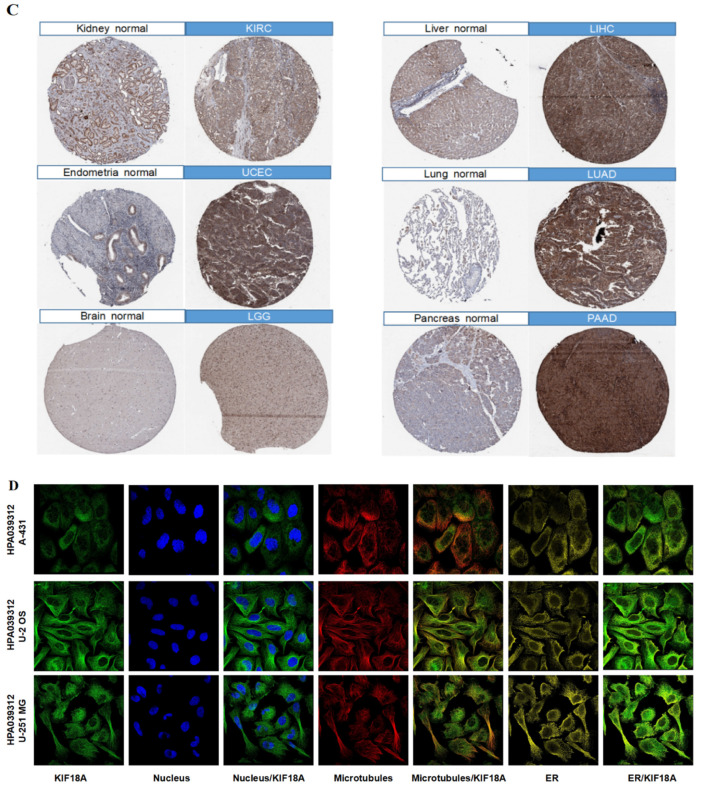
KIF18A expression in pan-cancer from different databases. (**A**) KIF18A expression in pan-cancer in TIMER. (**B**) KIF18A expression in cancers in TCGA + GTEx. (**C**) KIF18A expression in KIRC, LIHC, UCEC, LUAD, LGG, and PAAD from the HPA database. (**D**) Immunofluorescence staining of the subcellular localization of KIF18A in HPA database. (*, *p* < 0.05; **, *p* < 0.01; ***, *p* < 0.001, ns: no statistical differences).

The TIMER database was used to analyze the expression of KIF18A in pan-cancer and normal tissues. The results suggested that KIF18A expression was up-regulated in 16 cancers compared with normal tissues, including BLCA, BRCA, CESC, CHOL, COAD, ESCA, GBM, HNSC, KIRC, KIRP, LIHC, LUAD, LUSC, READ, STAD, and UCEC (Figure 1A). Since some tumors in the TIMER had no normal tissue, we combined TCGA with GTEx to study KIF18A expression in 33 tumors and found that KIF18A expression was up-regulated in 27 tumors compared with corresponding normal tissues. However, the expression of KIF18A was low in LAML and TGCT relative to normal tissues (Figure 1B). Based on the HPA database, KIF18A was highly expressed in KIRC, LIHC, UCEC, LUAD, LGG, and PAAD (Figure 1C), and KIF18A subcellular localization was obtained by immunofluorescence localization of the nuclei, microtubules, and ER in A-431, U-2OS, and U-251 MG cells. KIF18A was primarily located in microtubules and cytosols. In U-2 OS cells, KIF18A was located not only in microtubules and cytoplasm but also in the cyber-kinetic bridge (Figure 1D).

### 3.2. Relationship between KIF18A and Clinicopathological Data

**Figure 2:**
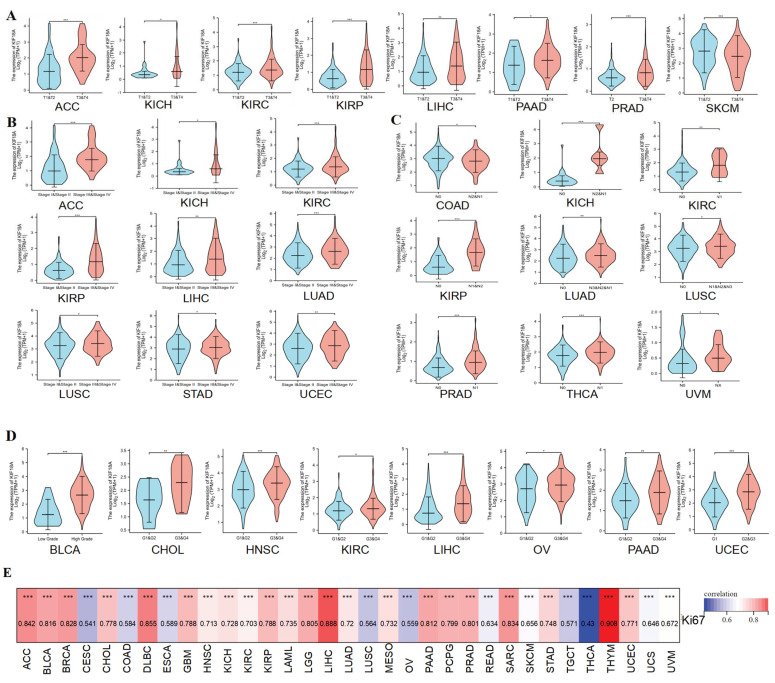
The relationship of KIF18A expression in cancers with clinical features. (**A**) The relationship of KIF18A expression to T stage in ACC, KICH, KIRC, KIRP, LIHC, PAAD, PRAD, and SKCM. (**B**) The relationship of KIF18A expression to the pathological stage in ACC, KICH, KIRC, KIRP, LIHC, LUAD, LUSC, STAD, and UCEC. (**C**) The relationship of KIF18A expression to N stage in COAD, KICH, KIRC, KIRP, LUAD, LUSC, PRAD, THCA, and UVM. (**D**) The relationship of KIF18A expression to histologic grade in BLCA, CHOL, HNSCC, KIRC, LIHC, OV, PAAD, and UCEC. (**E**) Pan−cancer correlation between KIF18A and Ki67 (*, *p* < 0.05; **, *p* < 0.01; ***, *p* < 0.001).

Subsequently, we explored the association between KIF18A and pathological characteristics. For the T stage, KIF18A had a higher expression in the higher T stage for ACC, KICH, KIRC, KIRP, LIHC, PAAD, and PRAD, and a lower expression in the higher T stage for SKCM (Figure 2A). KIF18A was highly expressed in the advanced stage of ACC, KICH, KIRC, KIRP, LIHC, LUAD, LUSC, STAD, and UCEC (Figure 2B). KIF18A expression in KICH, KIRC, KIRP, LUAD, LUSC, PRAD, THCA, and UVM was higher in patients with lymph node metastasis than without lymph node metastasis. However, the KIF18A expression level in COAD was lower in patients with lymph node metastasis than in patients without lymph node metastasis (Figure 2C). In BLCA, CHOL, HNSC, KIRC, LIHC, OV, PAAD, and UCEC, KIF18A was more highly expressed at higher histological gradings (Figure 2D). We found that KIF18A was moderately to highly correlated with the Ki67 index in 33 tumors (R > 0.4) (Figure 2E). Furthermore, the ROC curve estimated the diagnostic significance of KIF18A in 33 tumors. We discovered a high diagnostic value of KIF18A expression in 20 kinds of cancers (AUC > 0.8) (Figure S1), indicating that KIF18A can play a crucial role in tumor diagnosis.

### 3.3. Prognostic Value of KIF18A

**Figure 3:**
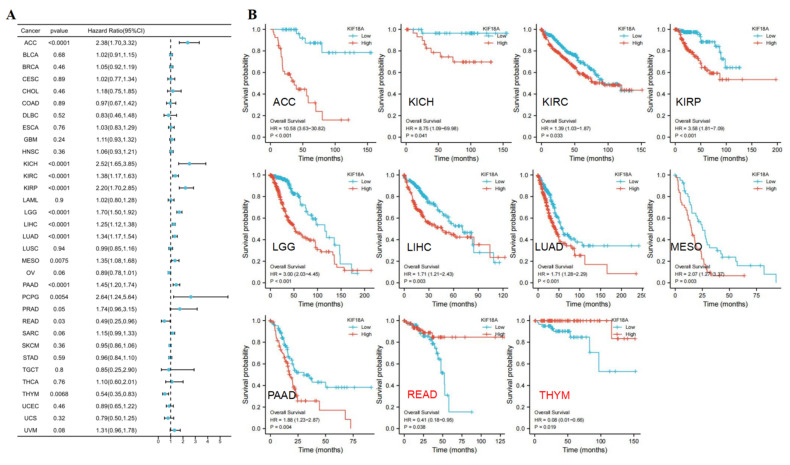

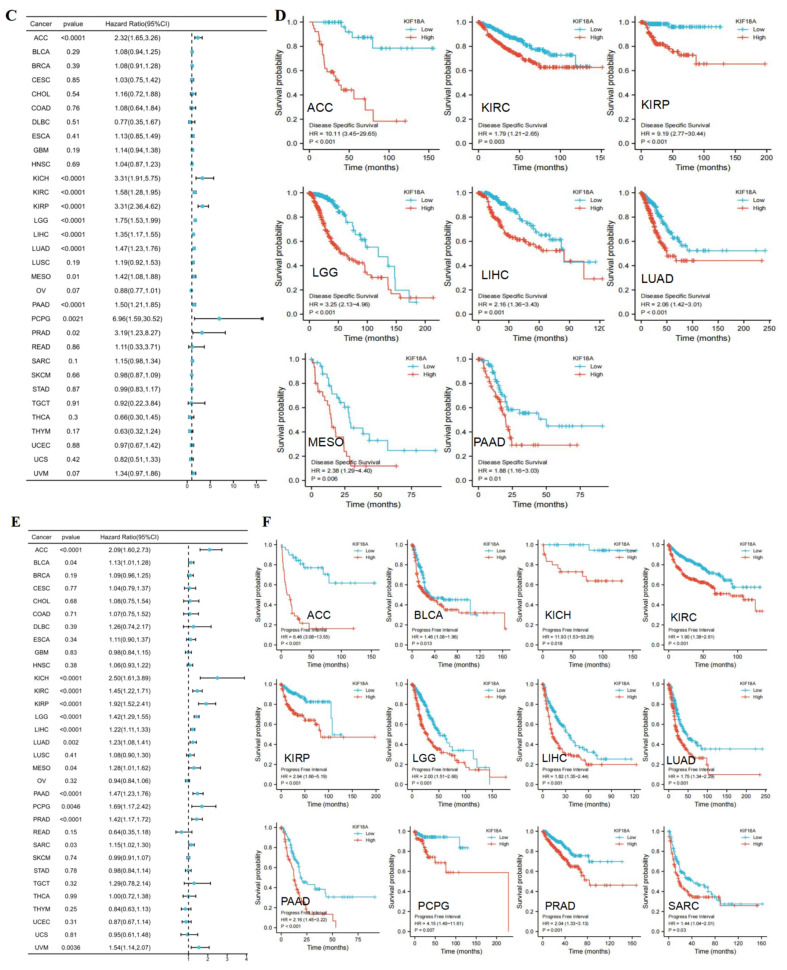
The univariate regression and Kaplan−Meier curves for OS (**A**,**B**), DSS (**C**,**D**), and PFI (**E**,**F**) in pan−cancer.

We chose OS, DSS, and PFI to investigate the prognosis of KIF18A in pan-cancer. High KIF18A expression had unfavorable OS in ACC, KICH, KIRC, KIRP, LGG, LIHC, LUAD, MESO, and PAAD. However, high expression of KIF18A had favorable OS in READ and THYM (Figure 3A,B). As for DSS, KIF18A high expression had shorter DSS in ACC, KIRC, KIRP, LGG, LIHC, LUAD, MESO, and PAAD (Figure 3C,D). The results displayed that KIF18A overexpression had shorter PFI in ACC, BLCA, KICH, KIRC, KIRP, LGG, LIHC, LUAD, PAAD, PCPG, PRAD, and SARC (Figure 3E,F). Therefore, KIF18A overexpression in ACC, KIRC, KIRP, LGG, LIHC, LUAD, and PAAD had shorter OS, DSS, and PFI. Moreover, the time-dependent ROC curve indicated that the 1-, 3-, and 5-year OS of KIF18A were above 0.6 in ACC, KIRC, KIRP, LGG, LIHC, LAUD, and PAAD (Figure S2). These findings suggested that KIF18A was an independent prognostic marker for ACC, KIRC, KIRP, LGG, LIHC, LUAD, and PAAD.

### 3.4. Correlation between KIF18A and Immune Cell Infiltration

**Figure 4:**
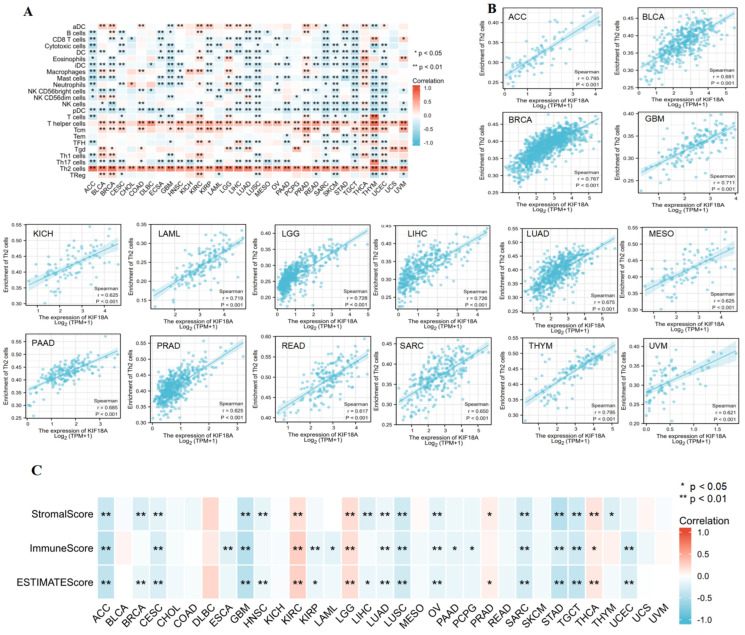
Correlation between KIF18A and immune cells in pan−cancer. (**A**) Heat map of KIF18A expression correlation with 24 tumor infiltrating cells. (**B**) The correlation between KIF18A expression and Th2 cells level in ACC, BLCA, BRCA, GBM, KICH, LAML, LGG, LIHC, LUAD, MESO, PAAD, PRAD, READ, SARC, THYM, and UVM. (**C**) The heat map of the correlation between KIF18A expression and the stromal score, immune score.

We explored the association between KIF18A expression and the infiltration of 24 kinds of immunocytes using Spearman correlations. KIF18A was positively related to Th2 infiltration levels in 33 cancers, and the correlation coefficient was greater than 0.6 in 16 cancers (Figure 4A,B). KIF18A expression had a positive association with T helper cells in 31 cancers. KIF18A was positively or negatively correlated with the infiltration of 22 other immune cells in a majority of the cancers. Afterwards, ESTIMATE algorithms were utilized to calculate the relationship of KIF18A with stromal score and immune score for 33 cancer types. KIF18A expression was negatively linked with stromal score and immune score in ACC, CESC, GBM, LUAD, LUSC, OV, SARC, STAD, and TGCT, but positively associated with stromal score and immune score in KIRC, LGG, and THCA. KIF18A expression had a positive or negative correlation with stromal score or immune score in some cancers. There was no relationship between KIF18A expression with stromal score and immune score of BLCA, CHOL, COAD, DLBC, KICH, MESO, READ, SKCM, UCS, and UVM (Figure 4C).

### 3.5. Relationship between KIF18A and Immune Regulatory Genes

**Figure 5:**
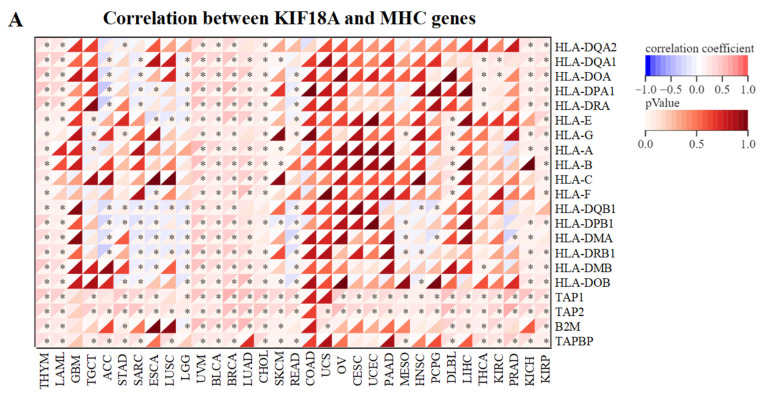

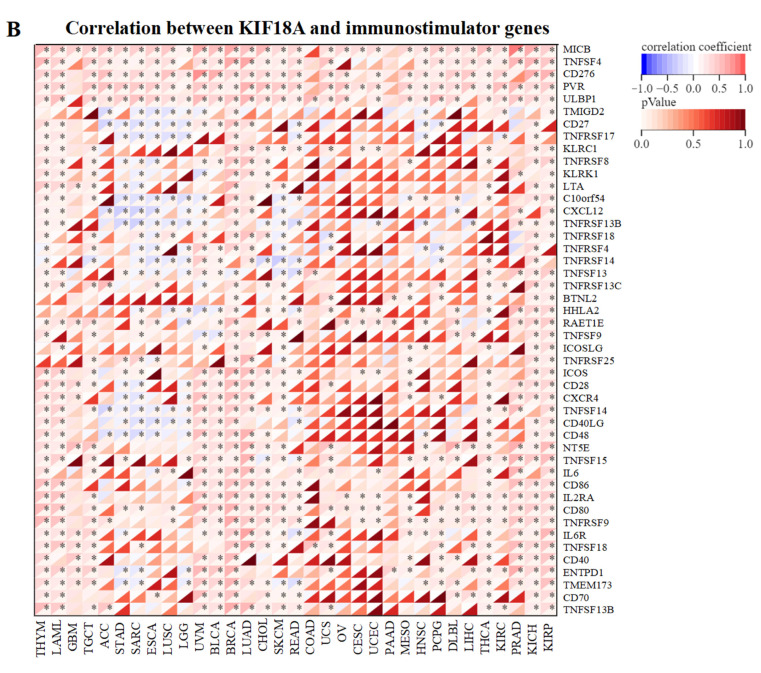

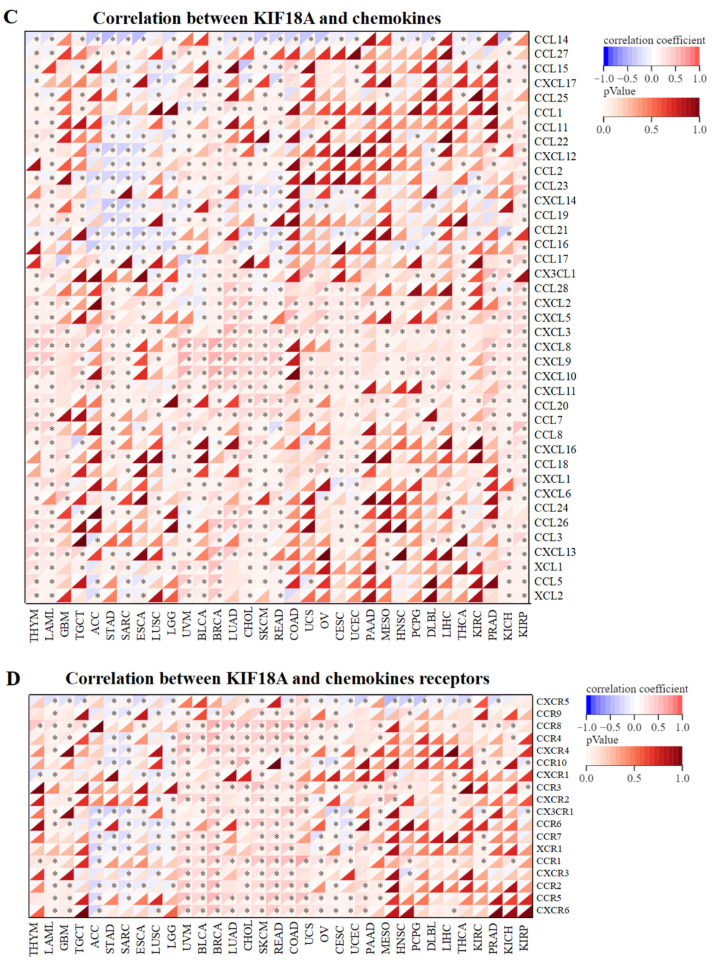

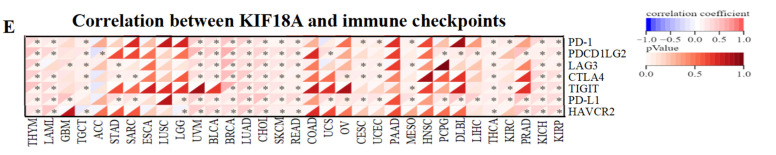
Correlation analysis between KIF18A and immune regulatory genes in pan-cancer. (**A**)Correlation between KIF18A and MHC genes. (**B**) Correlation between KIF18A and immunostimulator genes. (**C**) Correlation between KIF18A and chemokines. (**D**) Correlation between KIF18A and chemokines receptors. (**E**) Correlation between KIF18A and immune checkpoints. * *p* < 0.05.

We also investigated the relationship between KIF18A and immune regulatory genes in 33 tumors, including MHC, immunostimulator genes, chemokines, and chemokines receptors. In some cancers, KIF18A expression was significantly and positively correlated with immune regulatory genes(Figure 5A–D). Moreover, we found that KIF18A had a positive correlation with immune checkpoints, such as PD-1, PD-L1, CTLA4, TIGIT, LAG3, HAVCR2, and PDCD1LG2 in a great many cancers (Figure 5E).

### 3.6. Mutation Analysis of KIF18A

**Figure 6:**
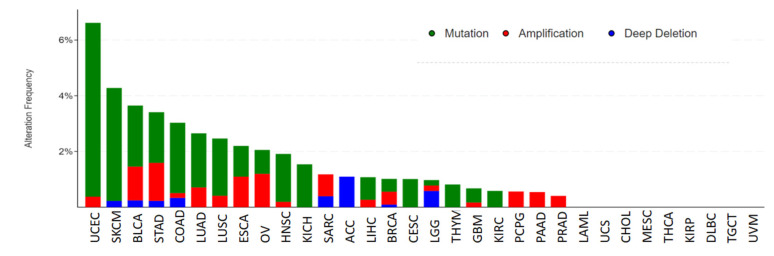
Genetic alterations of KIF18A in pan-cancer using the cBioPortal.

We discussed KIF18A genetic alterations in pan-cancer using the cBioPortal database. The frequency of genetic variation of KIF18A was the highest in UCEC (6.62%) and was mainly in the form of mutation. The second and the third highest frequency of KIF18A occurred in SKCM (4.42%) and BLCA (3.65%) and was also mainly in the form of mutation (Figure 6).

### 3.7. Relationship between KIF18A and TMB, MSI, MMR Genes

**Figure 7:**
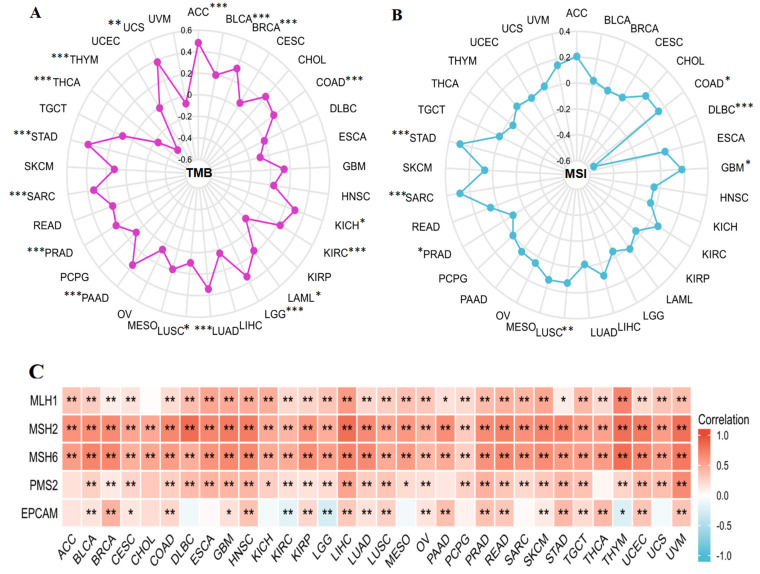
The correlation of KIF18A expression with TMB (**A**), MSI (**B**), and MMRs (**C**). (* *p* < 0.05, ** *p* < 0.01, *** *p* < 0.001).

We also performed the correlation analysis between KIF18A expression and TMB and MSI, which were significantly linked with immune checkpoint inhibitor (ICIs) sensitivity. A significant positive correlation was observed between KIF18A and TMB in ACC, BLCA, BRCA, COAD, KICH, KIRC, LAML, LGG, LUAD, LUSC, PAAD, PRAD, SARC, STAD, and UCS and a negative correlation with THCA and THYM (Figure 7A). KIF18A displayed a positive association with MSI in COAD, GBM, LUSC, SARC, and STAD and a negative association in DLBC and PRAD (Figure 7B). Moreover, KIF18A expression was positively linked with MLH1, MSH2, MSH6, PMS2, and EPCAM in most cancers, but was negatively correlated with EPCAM in KIRC, LGG, and THYM (Figure 7C).

### 3.8. Relationship between KIF18A Expression and DNA Methylation and RNA Methylation

**Figure 8:**
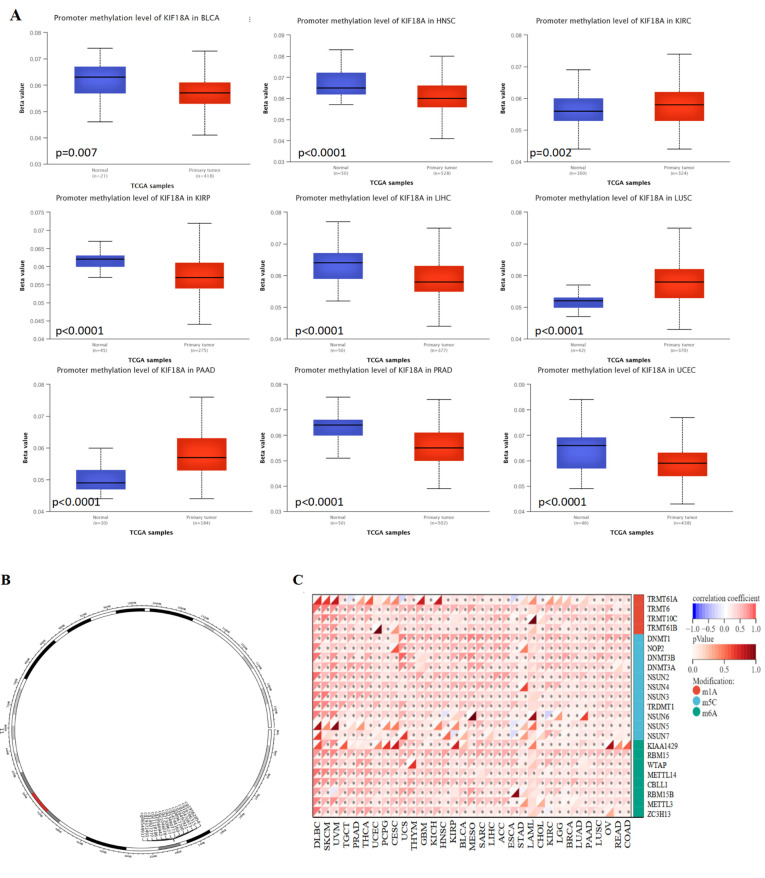
Relationship of KIF18A with methylation and methyltransferase. (**A**) Promoter methylation level of KIF18A in BLCA, HNSCC, KIRC, KIRP, LIHC, LUSC, PAAD, PRAD, and UCEC. (**B**) Chromosomal distribution of the methylation probes associated with KIF18A. (**C**) The correlation between KIF18A expression and m^1^A, m^5^C, m^6^A regulatory genes. * *p* < 0.05.

We examined the DNA methylation levels of KIF18A for various tumors using the UALCAN database, and the results showed that the methylation level of KIF18A in BLCA, HNSC, KIRP, LIHC, PRAD, and UCEC was lower than that in normal tissues; this may be an explanation for the high KIF18A expression in these tumors. Increased methylation levels were observed in KIRC, LUSC, and PAAD (Figure 8A). MethSurv was used for DNA methylation level analysis and survival analysis for each CpG site of KIF18A. As shown Figure 8B, KIF18A had 14 methylation probes, such as cg19562854, cg16786315, cg10470873, cg16497921, cg08967200, cg14744160, cg23490773, cg27309677, cg21046078, cg20566942, cg21118186, cg14927277, cg19372491, and cg06544937. Hypermethylated cg08967200 suggested a good prognosis in ACC and a poor outcome in BLCA, HNSC, KIRC, and LIHC. The prognosis of hypermethylation of cg10470873 was good in ACC, KIRC, and LGG, but poor in KIRP, SKCM, STAD, and UCEC. Cg19562854 hypermethylation had a good outcome in ACC, LGG, MESO, and PAAD, but a poor outcome in BLCA, COAD, KIRP, and LIHC. The hypermethylation of cg16497921 was related to the good prognosis of KIRC and SARC but was correlated with the bad prognosis of CESC, KIRP, LAML, LIHC, and UCEC. The hypermethylation of cg16786315 in COAD, ESCA, LIHC, PAAD, and UCEC indicated a poor prognosis. However, the hypomethylation of cg16786315 indicated a poor prognosis in KIRC, SARC, and SKCM (Table S1). KIF18A might have affected the prognosis of cancer patients through methylation. Moreover, KIF18A was positively correlated to m6A, m5C, and m1A regulatory genes in most cancers. However, in LAML, KIF18A was not related to m1A regulatory genes and was related to a small number of m5C and m6A regulatory genes (Figure 8C). KIF18A may play a carcinogenic role by affecting the expression level of RNA methylation regulatory genes.

### 3.9. Single-Cell Functional Analysis of KIF18A

**Figure 9:**
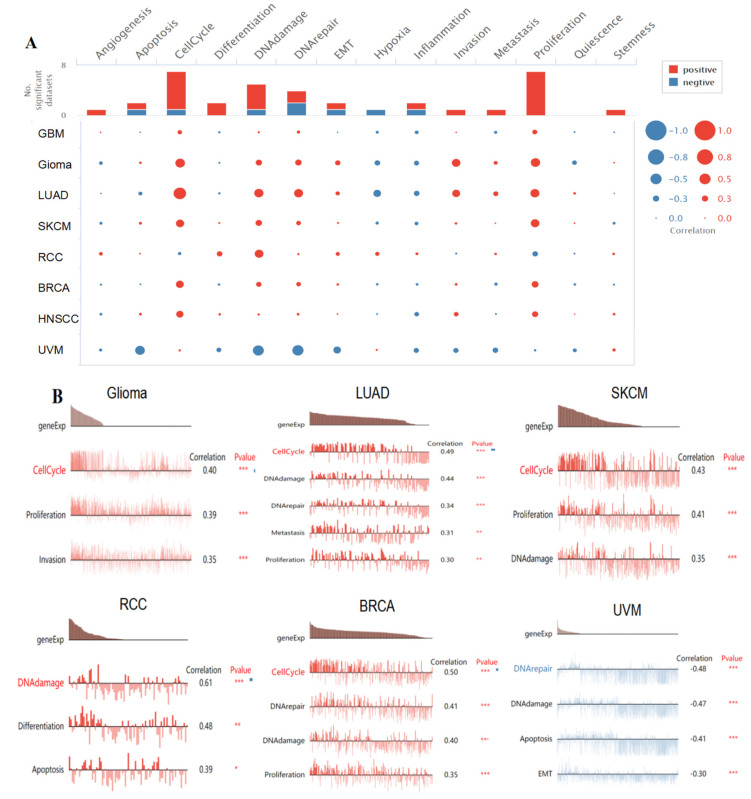
The function of KIF18A in single-cell functional analysis from the CancerSEA database. (**A**) Functional status of KIF18A in different human cancers. (**B**) Correlation analysis between functional status and KIF18A in glioma, LUAD, SKCM, RCC, BRCA, and UVM. (* *p* < 0.05, ** *p* < 0.01, *** *p* < 0.001).

To further study the latent role of KIF18A in tumors, we investigated the function of KIF18A at the single-cell level using CancerSEA (Figure 9A). The findings displayed that KIF18A was positively linked with cell cycle, proliferation, and invasion of glioma. In LUAD, KIF18A had a positive relationship with cell cycle, DNA damage, DNA repair, metastasis, and proliferation. KIF18A expression and cell cycle, proliferation, and DNA damage in SKCM were positively correlated. KIF18A was positively related to DNA damage, differentiation, apoptosis in RCC and cell cycle, DNA repair, DNA damage, and proliferation in BRCA. However, there was a negative relationship between KIF18A and DNA repair, DNA damage, apoptosis, and EMT in UVM (Figure 9B).

### 3.10. Drug Sensitivity Analysis

**Figure 10:**
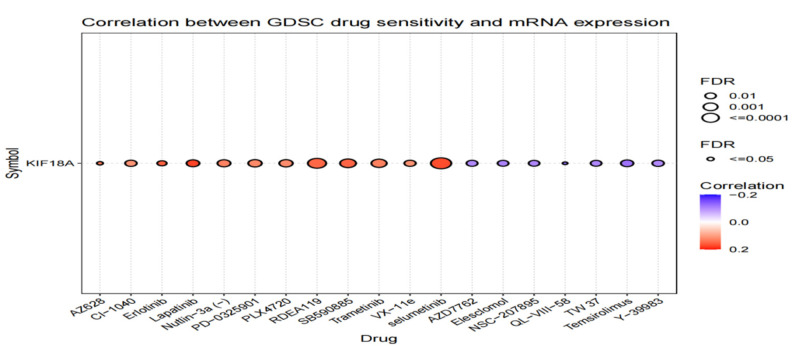
The associations of KIF18A expression and drug sensitivity base on GSCALite.

We used GSCALite to investigate the drug sensitivity of KIF18A expression in tumors. KIF18A expression was negatively associated with 50% inhibitory concentration (IC50) values of AZD7762, Elesclomol, NSC-207895, QL-VIII58, TW37, Temsirolimus, and Y-39983. Moreover, there was a positive correlation between KIF18A expression and IC50 values of 12 types of drugs, such as AZ628, CI-1040, Eriotinib, Lapatinib, Nutlin-3a, PD-0325901, PLX4720, RDEA119, SB590885, Trametinib, VX-11e, and selumetinib (Figure 10).

### 3.11. Protein Interaction Network and Enrichment Analysis of KIF18A 

**Figure 11:**
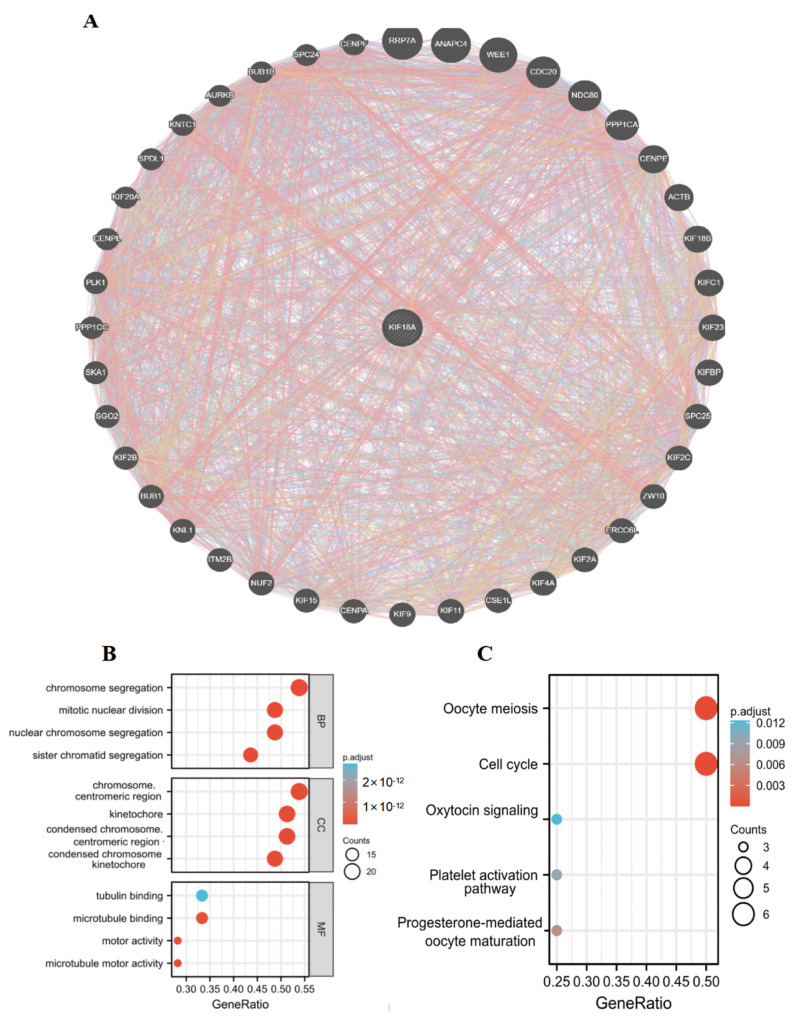

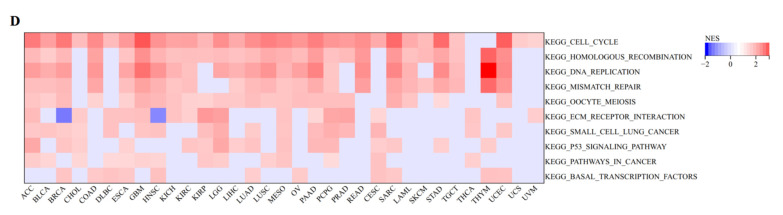
(**A**) Forty genes co-expressed with KIF18A in GeneMANIA. (**B**) GO enrichment analysis of KIF18A related genes. (**C**) KEGG enrichment analysis of KIF18A-related genes. (**D**) GSEA enrichment results.

To further study the potential effect of KIF18A in tumorigenesis, 40 genes related to KIF18A were extracted from the GeneMANIA database to perform GO and KEGG enrichment analyses (Figure 11A). Biological process (BP) enrichment analysis showed that KIF18A-related genes were mainly involved in chromosome segregation, mitotic nuclear division, nuclear chromosome segregation, and sister chromatid segregation. Molecular function (MF) enrichment analysis showed that the role of KIF18A in tumor pathogenesis was related to tubulin binding, microtubule binding, microtubule motor activity, and motor activity. We found that KIF18A-related genes were enriched in the chromosome, centromeric region, kinetochore, and condensed chromosome kinetochore in cellular component (CC) enrichment analysis (Figure 11B). In addition, KEGG pathway analysis showed that KIF18A participated in some pathways, such as cell cycle, oocyte meiosis, progesterone-mediated oocyte maturation, etc (Figure 11C). Subsequently, we applied GSEA to determine the biologic functions of KIF18A in tumors. We discovered that KIF18A mainly participated in the cell cycle, homologous recombination, and DNA replication of most cancers (Figure 11D). These results indicated the molecular mechanism of KIF18A in tumorigenesis.

### 3.12. The Verification of the High Expression of KIF18A by IHC in Some Tumors

**Figure 12:**
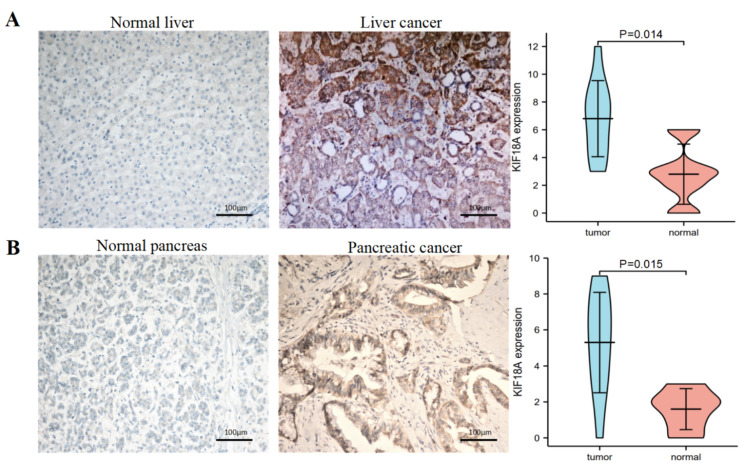
KIF18A protein expression in tumors and normal tissues by immunohistochemistry (EnVision; original magnification, ×200). (**A**) The expression of KIF18A protein was higher in liver cancer than normal liver tissue. (**B**) The expression of KIF18A protein was higher in pancreatic cancer than normal pancreas tissues.

To further clarify the difference in the protein expression of KIF18A in tumor tissues and normal tissues, we selected ten cases of human liver cancer and ten cases of pancreatic cancer and their corresponding normal samples from the Department of Pathology, Beijing Ditan Hospital, Capital Medical University for an IHC study. The semi-quantitative scoring of IHC demonstrated that the KIF18A protein was significantly overexpressed in LIHC (Figure 12A) and PAAD (Figure 12B) compared with corresponding normal tissues. The overexpression of KIF18A in tumors increased the degree of credibility for KIF18A as a new target in therapies for these cancers.

### 3.13. KIF18A Is Regarded as an Independent Marker in LIHC and PAAD 

**Figure 13:**
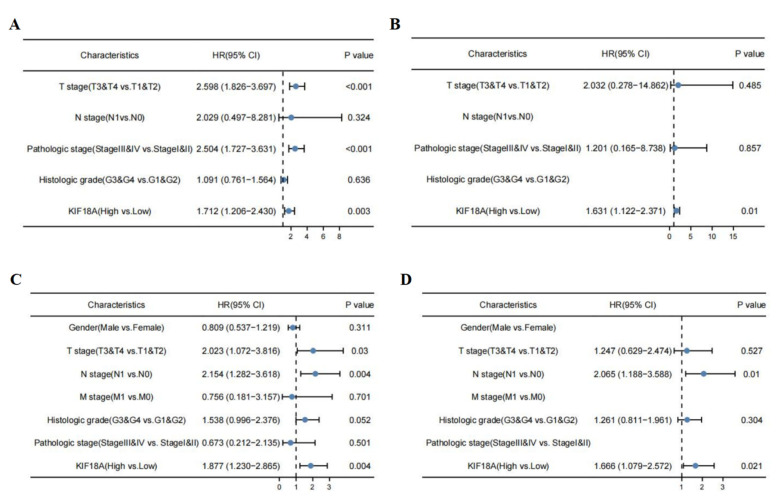
Univariate and multivariate analyses for OS in LIHC (**A**,**B**) and PAAD (**C**,**D**).

We used logistic regression analysis to estimate the correlation between KIF18A expression and clinical characteristics in LIHC and PAAD from TCGA database. Univariate analysis confirmed that T stage, pathological stage and KIF18A expression were important prognostic factors of OS in LIHC. KIF18A expression were identified as independent prognostic factors in LIHC by multivariate analysis (Figure 13A,B). In terms of PAAD, univariate analysis showed that OS was statistically significant and influenced by T stage, N stage, and KIF18A expression, and further multivariate analysis demonstrated that N stage and KIF18A expression were regarded as independent prognostic factors (Figure 13C,D). In conclusion, KIF18A expression was considered as a marker for an unfavorable prognosis on LIHC and PAAD by univariate and multivariate analysis.

## 4. Discussion

KIF18A, as a member of the Kinesin family, is a crucial regulating factor of chromosome arrangement during mitosis [38,39]. Recent studies have shown that KIF18A participates in the pathogenesis and progression of various tumors, such as colorectal cancer, breast cancer, prostate cancer, liver cancer, head and neck squamous cell carcinoma, lung adenocarcinoma, gastric cancer, glioma, and clear cell renal carcinoma, and is also related to these tumors’ survival and prognosis [8,10,11,12,13,14,15,17,18,19]. We used a variety of databases from TCGA, GTEx, UALCAN, cBioportal, and others to reveal the molecular characteristics of KIF18A in 33 tumors from an overall perspective, including gene expression, prognosis, gene alterations, immune infiltration, DNA methylation, RNA methylation, and drug sensitivity to clarify its role in the development and potential regulatory pathways of different tumors.

Our work confirmed the significant overexpression of KIF18A in 27 cancers from TCGA and GTEx databases. We performed IHC on liver cancer and pancreatic cancer to confirm the expression of KIF18A protein in cancers and normal tissue samples. The expression of KIF18A protein was higher in LIHC and PAAD compared to normal tissues. These findings suggest an important role of KIF18A in different cancers. KIF18A’s high expression indicated poor OS, DSS, and PFI in ACC, KIRC, KIRP, LGG, LIHC, LUAD, and PAAD. Moreover, KIF18A was an independent biomarker in LIHC and PAAD according to the univariate and multivariate regression analyses. These results are consistent with earlier findings [13,14,15,16,17]. The expression of KIF18A in ACC and PAAD was first reported. In several tumors, KIF18A had higher expression in higher T stages, higher clinical stages, higher N stages, and higher histological grades. This study showed that, in 33 kinds of tumors, KIF18A was highly positively correlated with the Ki67 index, indicating that KIF18A expression level increased with the increase in the Ki67 proliferation index. Therefore, it is speculated that KIF18A may accelerate tumor cell division by interfering with the cell cycle, resulting in the formation of tumors. These findings show that KIF18A can be used as a latent marker for predicting the outcomes of tumors.

The TME is the growth environment of the tumor and invasive immune and stromal cells. More and more studies have proven that immunocyte infiltration is a crucial element in tumor progress and immunotherapy [40]. Therefore, it is vitally important to discuss the relationship between KIF18A and tumor-associated immune cell infiltration. Interestingly, KIF18A expression had a positive association with Th2 for 33 cancers in the TCGA database. This phenomenon may be due to the excessive secretion of Th2 cytokines in tumor tissues. Th2 dominance suppresses anti-tumor immunity so that tumor cells can escape immunological surveillance and immune attack, resulting in tumors. Moreover, stromal cells and immune cells, as two non-tumor components of the TME, have an important role in tumor biology [41]. Research has shown that immune scores can be used as an indicator for the survival, recurrence, metastasis, and drug resistance of cancer patients [42]. We found that KIF18A was positively correlated with a stromal and immune score in three cancers and negatively in nine cancers, suggesting that KIF18A interacted with tumor cells and immune cells in tumors. This finding provided a new perspective for the development of effective therapies. Immune checkpoint genes are important targets for immune checkpoint inhibitors (ICIs) in the treatment of various cancers [43]. At present, ICIs are an effective anti-cancer immunotherapy method [44]. Furthermore, KIF18A expression was positively correlated with many common immune checkpoints, including PD-L1, PD-1, CTLA4, TIGIT, HAVCR2, PDCD1LG2, and LAG3, indicating that KIF18A may be a new target for tumor immunotherapy. In addition, our research demonstrated that KIF18A was significantly associated with immune regulatory genes. These results indicate that KIF18A expression was closely linked to the immune infiltration of tumor cells, influencing the prognosis of tumor patients, and this provides a new target for the development of immunosuppressants.

TMB and MSI are reliable biomarkers for prognosis in various cancers and predictors of many tumors’ immunotherapeutic effect [45,46]. Tumors with high TMB and high MSI, have better response to immunotherapy [47,48,49]. Our results demonstrated that KIF18A expression was significantly positively correlated with TMB in 15 tumors and with MSI in 5 tumors. Interestingly, KIF18A expression was positively related to TMB and MSI in COAD and LUSC. Therefore, we speculated that the high expression of KIF18A in COAD and LUSC will have greater survival benefits after immunotherapy. KIF18A can thus be used as a new drug target for anti-cancer immunotherapy.

DNA methylation is one of the most common epigenetic modifications, which plays an important role in gene expression, genomic stability, and tumorigenesis. It has been shown that aberrant DNA methylation may accelerate tumor development by regulating cell proliferation, thereby inducing apoptosis or senescence [50]. Using the UALCAN tool, we observed that the KIF18A promoter methylation level was significantly decreased in BLCA, HNSC, KIRP, LIHC, PRAD, and UCEC compared to normal tissues and increased in KIRC, LUSC, and PAAD. RNA methylation is involved in tumorigenesis, development, and prognosis [51]. In our work, KIF18A expression was positively interrelated with regulatory genes of RNA methylation for several cancers. These findings indicated that KIF18A might promote tumorigenesis through RNA methylation.

We used CancerSEA and GSEA to perform pan-cancer functional analyses of KIF18A. Single-cell function analysis showed that KIF18A was positively related to cell cycle and proliferation in some tumors. Recent studies have shown that the inhibition of KIF18A expression in HCC could reduce cell cycle-related protein expression levels, and KIF18A might promote HCC cell proliferation through the cell cycle signaling pathway [13]. GSEA analysis of KIF18A revealed that KIF18A was primarily involved in the cell cycle, homologous recombination, and DNA replication of some cancers. This is consistent with previous research [8]. However, the interaction between KIF18A and the cell cycle is not completely comprehended and needs to be investigated further in future work. Furthermore, the correlation between KIF18A mRNA and anticancer drug sensitivity was examined. The overexpression of KIF18A was negatively correlated with the IC50 values of seven types of the drug through the GDSC database, including AZD7762, Elesclomol, NSC-207895, TW-37, Temsirolimus, Y-39983, and QL-VIII-58. These drugs had the potential to prevent cancer progression. This finding would help guide clinical drug selection and patient prognosis.

Although the effect of KIF18A in pan-cancer was described, some limitations should not be overlooked. Firstly, all the analyses were based on bioinformatics analysis, and KIF18A expression was only confirmed by IHC in some tumors. Secondly, our study did not investigate the molecular mechanism of KIF18A in cancers. Further studies on the mechanism of KIF18A expression in tumors are needed in the future.

## 5. Conclusions

Our research systematically investigated that KIF18A expression was significantly linked with clinical features, prognosis, mutational status, DNA methylation, RNA methylation, TME, TMB, MSI, MMRs, and drug sensitivity in several cancers, which helped us to better understand the potential role of KIF18A in pan-cancer.

## Figures and Tables

**Table 1:** The abbreviations and corresponding full names of 33 cancers.

Cancer Type	Abbreviation
adrenocortical carcinoma	ACC
bladder urothelial carcinoma	BLCA
breast invasive carcinoma	BRCA
cervical squamous cell carcinoma and endocervical adenocarcinoma	CESC
cholangiocarcinoma	CHOL
colon adenocarcinoma	COAD
lymphoid neoplasm diffuse Large B-cell lymphoma	DLBC
esophageal carcinoma	ESCA
glioblastoma multiforme	GBM
head and neck squamous cell carcinoma	HNSC
kidney chromophobe	KICH
kidney renal clear cell carcinoma	KIRC
kidney renal papillary cell carcinoma	KIRP
acute myeloid leukemia	LAML
brain lower grade glioma	LGG
liver hepatocellular carcinoma	LIHC
lung adenocarcinoma	LUAD
lung squamous cell carcinoma	LUSC
mesothelioma	MESO
ovarian serous cystadenocarcinoma	OV
pancreatic adenocarcinoma	PAAD
pheochromocytoma and paraganglioma	PCPG
prostate adenocarcinoma	PRAD
rectum adenocarcinoma	READ
sarcoma	SARC
skin cutaneous melanoma	SKCM
stomach adenocarcinoma	STAD
testicular germ cell tumors	TGCT
thyroid carcinoma	THCA
thymoma	THYM
uterine corpus endometrial carcinoma	UCEC
uterine carcinosarcoma	UCS
uveal melanoma	UVM

## Data Availability

The original manuscript contained in the research report is included in the article/Supplementary Materials. Further inquiries can be made directly to the corresponding author.

## Supplementary Materials

The following supporting information can be downloaded at: https://www.mdpi.com/article/10.3390/biom13020326/s1. Figure S1: The ROC curve for KIF18A in ACC, BRCA, CHOL, COAD, ESCA, GBM, HNSC, LAML, LGG, LIHC, LUSC, OV, PAAD, STAD, UCEC, UCS, BLCA, LUAD, KIRC, and TGCT. Figure S2: The time-dependent ROC curve of 1-, 3-, 5-year survival in ACC, KIRC, KIRP, LGG, LIHC, LUAD, and PAAD. Table S1: Relationship between KIF18A methylated CpG and survival.
