# Supplementary material for: Comprehensive Pan-Cancer Analysis of KIF18A as a Marker for Prognosis and Immunity
Source: Biomolecules. 2023 Feb 8;13(2):326. doi: 10.3390/biom13020326 (PMC9953516; doi:10.3390/biom13020326)
Supplement: Supplementary file 1 [file biomolecules-13-00326-s001.zip › supplementary +e++.pdf]

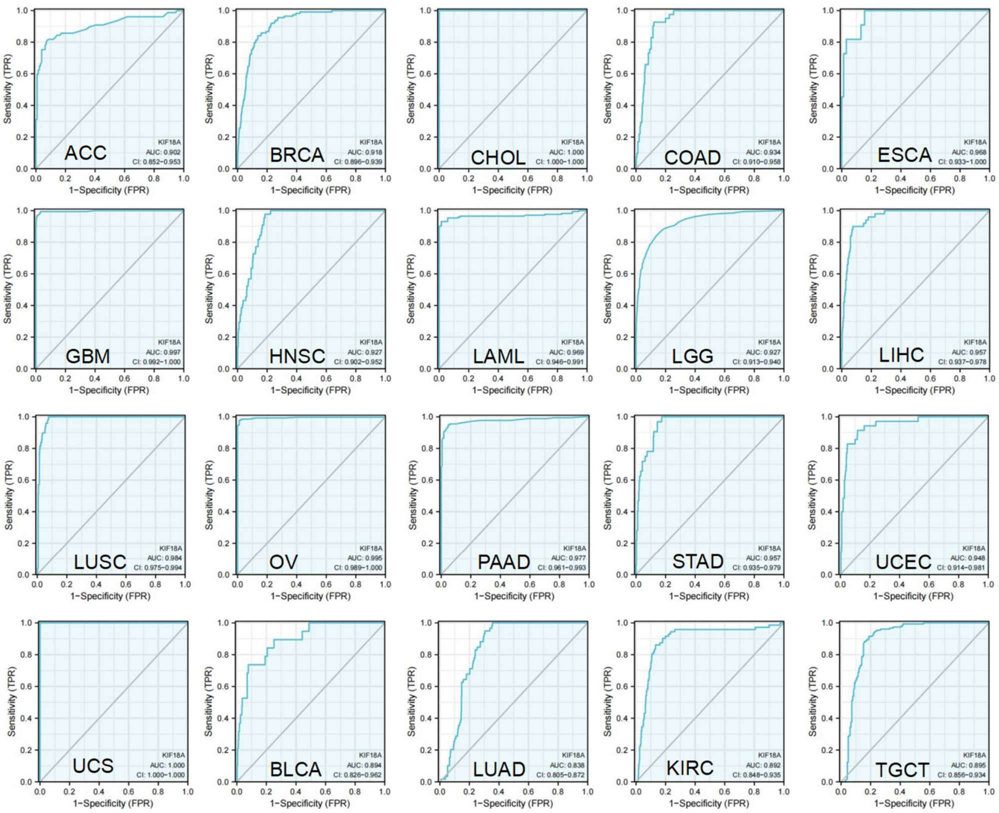

Figure S1. The ROC curve for KIF18A in ACC, BRCA, CHOL, COAD, ESCA, GBM, HNSC, LAML, LGG, LIHC, LUSC, OV, PAAD, STAD, UCEC, UCS, BLCA, LUAD, KIRC and TGCT.

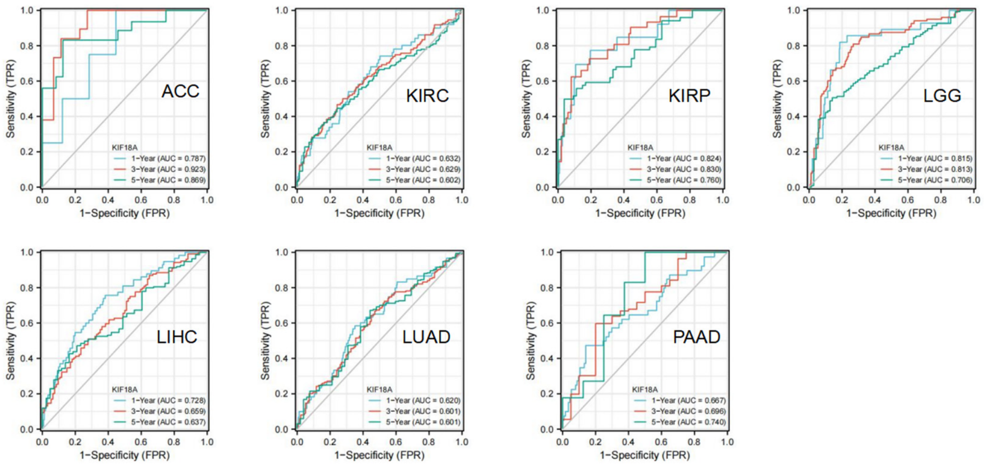

Figure S2. The time-dependent ROC curve of 1-,3-,5-year in ACC, KIRC, KIRP, LGG, LIHC, LUAD and PAAD.

Table S1. Relationship between KIF18A methylated CpG and survival.

	CpG	HR	*P* value
**ACC**	cg08967200	0.426	0.033
	cg10470873	0.432	0.03
	cg19562854	0.454	0.048
**BLCA**	cg08967200	1.427	0.029
	cg19562854	1.388	0.043
**CESC**	cg16497921	1.644	0.039
**COAD**	cg16786315	1.659	0.039
	cg19562854	1.641	0.046
**ESCA**	cg16786315	1.699	0.021
**HNSC**	cg08967200	1.315	0.044
**KIRC**	cg08967200	1.917	0.0012
	cg10470873	0.453	0.003
	cg16497921	0.596	0.038
	cg16786315	0.426	0.0018
**KIRP**	cg10470873	2.07	0.028
	cg16497921	4.434	0.013
	cg19562854	2.636	0.043
**LAML**	cg16497921	1.576	0.031
**LGG**	cg10470873	0.629	0.017
	cg19562854	0.632	0.018
**LIHC**	cg08967200	1.866	0.01
	cg16497921	1.557	0.012
	cg16786315	1.498	0.02
	cg19562854	1.734	0.0017
**MESO**	cg19562854	0.555	0.014
**PAAD**	cg16786315	1.514	0.044
	cg19562854	0.572	0.011
**SARC**	cg16497921	0.666	0.049
	cg16786315	0.572	0.0073
**SKCM**	cg10470873	1.314	0.045
	cg16786315	0.717	0.039
**STAD**	cg10470873	1.65	0.018
**UCEC**	cg10470873	2.065	0.022
	cg16497921	2.349	0.012
	cg16786315	2.197	0.013
